# The Wheat Gene *TaVQ14* Confers Salt and Drought Tolerance in Transgenic *Arabidopsis thaliana* Plants

**DOI:** 10.3389/fpls.2022.870586

**Published:** 2022-05-10

**Authors:** Xinran Cheng, Hui Yao, Zuming Cheng, Bingbing Tian, Chang Gao, Wei Gao, Shengnan Yan, Jiajia Cao, Xu Pan, Jie Lu, Chuanxi Ma, Cheng Chang, Haiping Zhang

**Affiliations:** ^1^College of Agronomy, Anhui Agricultural University, Key Laboratory of Wheat Biology and Genetic Improvement on Southern Yellow and Huai River Valley, Ministry of Agriculture and Rural Affairs, Hefei, China; ^2^National Key Laboratory for Crop Genetics and Germplasm Enhancement, Jiangsu Plant Gene Engineering Research Center, Nanjing Agricultural University, Nanjing, China

**Keywords:** wheat, salt, drought, subcellular localization, expression analysis, *TaVQ14*

## Abstract

Wheat is one of the most widely cultivated food crops worldwide, and the safe production of wheat is essential to ensure food security. Soil salinization and drought have severely affected the yield and quality of wheat. Valine-glutamine genes play important roles in abiotic stress response. This study assessed the effect of the gene *TaVQ14* on drought and salt stresses resistance. Sequence analysis showed that TaVQ14 encoded a basic unstable hydrophobic protein with 262 amino acids. Subcellular localization showed that TaVQ14 was localized in the nucleus. *TaVQ14* was upregulated in wheat seeds under drought and salt stress. Under NaCl and mannitol treatments, the percentage of seed germination was higher in Arabidopsis lines overexpressing *TaVQ14* than in wild-type lines, whereas the germination rate was significantly lower in plants with a mutation in the *atvq15* gene (a *TaVQ14* homolog) than in WT controls, suggesting that *TaVQ14* increases resistance to salt and drought stress in Arabidopsis seeds. Moreover, under salt and drought stress, Arabidopsis lines overexpressing *TaVQ14* had higher catalase, superoxide dismutase, and proline levels and lower malondialdehyde concentrations than WT controls, suggesting that *TaVQ14* improves salt and drought resistance in Arabidopsis by scavenging reactive oxygen species. Expression analysis showed that several genes responsive to salt and drought stress were upregulated in Arabidopsis plants overexpressing *TaVQ14*. Particularly, salt treatment increased the expression of *AtCDPK2* in these plants. Moreover, salt treatment increased Ca^2+^ concentrations in plants overexpressing *TaVQ14*, suggesting that *TaVQ14* enhances salt resistance in Arabidopsis seeds through calcium signaling. In summary, this study demonstrated that the heterologous expression of *TaVQ14* increases the resistance of Arabidopsis seeds to salt and drought stress.

## Introduction

Wheat (*Triticum aestivum* L.) is a widely cultivated grain crop and one of the three most important cereal grains worldwide (Brenchley et al., 2012). Wheat is a good energy source and provides essential nutrients such as protein, vitamins, and trace elements. Therefore, the safe production of wheat is crucial to ensure food security globally.

Droughts, soil salinization, and other environmental stresses are the main factors limiting the increase in wheat yield and quality. Resistance to drought and salinity is a complex biological trait involving multiple molecular, physiological, biochemical, and morphological changes (Zhou et al., 2018; Wang and Huang, 2019; Zelm et al., 2020). Therefore, identifying key genes and elucidating the mechanisms regulating crop resistance to drought and salinity stress are useful for increasing food production while allowing the sustainable management of ecological resources (Ingram and Bartels, 1996; Xiong et al., 2002; Jakab et al., 2005; Verslues et al., 2006; Yamaguchi-Shinozaki and Shinozaki, 2006).

Valine-glutamine-motif proteins have attracted increasing attention because of their interaction with WRKY transcription factors (Lai et al., 2011). A total of 34, 40, and 61 VQ proteins have been identified in Arabidopsis, rice, and maize, respectively (Cheng et al., 2012; Li N. et al., 2014; Wang et al., 2014). These proteins regulate plant growth and development and the response to biotic and abiotic stresses (Andreasson et al., 2005; Petersen et al., 2010; Wang et al., 2010; Fill and Petersen, 2011; Hu P. et al., 2013; Hu Y. et al., 2013; Kim et al., 2013; Li Y. et al., 2014; Wang et al., 2014; Pascal et al., 2014; Wang H. et al., 2015; Wang M. et al., 2015; Song et al., 2016; Li et al., 2020). For instance, *AtVQ14 (IKU1)* is strongly expressed in the embryo and endosperm and controls endosperm development and seed size. Given that the nutrients required for seed germination are affected by seed size, thus it also affects resistance to adverse environments (Wang et al., 2010). *AtVQ29* is involved in the photomorphogenesis of *Arabidopsis* seedlings. The hypocotyls of plants overexpressing *AtVQ29* are longer than those of wild-type (WT) plants under far-red light or low light and regulate flowering time (Li Y. et al., 2014). *AtVQ21 (MSK1)* transgenic plants positively regulate the resistance of the pathogen *Pseudomonas syringae* and negatively mediate the resistance of the pathogen *Botrytis cinerea* (Petersen et al., 2010; Fill and Petersen, 2011). *AtVQ22* improves JA-mediated disease resistance, mutant plants overexpressing this gene are more resistant to necrotizing pathogens, and transgenic lines were extremely sensitive to pathogen infection. In addition, the analysis of the rice transcriptome showed that *VQ22* expression increased after infection with *Magnaporthe grisea*, indicating that this gene plays an important role in disease resistance (Hu Y. et al., 2013). *AtCaMBP25* (*AtVQ15*) reduced osmotic stress during seed germination and growth in *Arabidopsis thaliana*. Under salt and osmotic stress, transgenic lines are highly sensitive to seed germination, growth, and development (Wang M. et al., 2015). Seed germination and seedling growth were inhibited in plants overexpressing *AtVQ9* under salt stress (Hu Y. et al., 2013). *ZmVQ54*, *ZmVQ19*, *OsVQ2*, *OsVQ16*, and *OsVQ20* were highly expressed under drought stress (Kim et al., 2013). *GmVQ6* and *GmVQ53* were highly expressed in roots and stems under low-nitrogen conditions. *Arabidopsis* lines overexpressing (Wang et al., 2014). *PeVQ28* were salt tolerant (Cheng et al., 2020). These data demonstrate that *VQ* genes have multiple roles in regulating plant growth and development and resistance to biotic and abiotic stresses.

Little is known about the functions of *VQ* genes in wheat. Our previous study showed that *TaVQ14* was related to salt stress response (Cheng et al., 2021). Therefore, *TaVQ14* was selected as the target gene for further functional analysis. First, the subcellular location of TaVQ14 was determined. Second, *Arabidopsis* plants overexpressing *TaVQ14* were obtained by genetic transformation, and molecular, physiological, and phenotypic analyses were carried out. This study elucidated the functions of VQ genes and provided useful information for genetically improving salt and drought resistance in wheat crops.

## Materials and Methods

### Experimental Materials and Stress Treatment

Wheat varieties Jing 411 (J411) and Hongmangchun 21 (HMC21) were provided by Shihe Xiao from the Chinese Academy of Agricultural Sciences, both of which were moderately salt and drought tolerant (Ren, 2012). Wheat seeds were treated with 300 mM NaCl or 300 mM mannitol. Samples were collected at 0, 4, 6, 10, 48, and 72 h, frozen in liquid nitrogen and stored immediately at −80°C.

Tobacco (*Nicotiana tabacum*) and Colombian ecotype *Arabidopsis* [Columbia-0 (Col-0), wild-type (WT)] seeds were provided by the State Key Laboratory of Crop Resistance of Anhui Agricultural University and were expanded and preserved in our laboratory. The seeds of WT and mutant *Arabidopsis* lines were cultivated in Murashige and Skoog (MS) medium at 24°C under a 16-h light/8-h dark cycle and transferred to square pots (diameter 6 cm) containing black soil and verstone (1:3, v/v) (Chen D. et al., 2017; Gao et al., 2017). Tobacco seeds were planted in square pots (6 cm in diameter) containing black soil and verstone (1:2, v/v).

### Bioinformatics Analysis of *TaVQ14*

*TaVQ14* sequences were obtained from the Ensembl Plants database. The isoelectric point (pI), molecular weight (MW), and other properties of TaVQ14 protein were analyzed using ExPASy (Cheng et al., 2018). The number of exons and introns of *TaVQ14* was analyzed using Gene Structure Display Server^1^ (Zhu et al., 2018). The phylogenetic analysis of *TaVQ14* was performed using MEGA 7.0 (Cheng et al., 2019a). The promoter region of *TaVQ14* was analyzed using PlantCARE (Toufighi et al., 2005; Cheng et al., 2019b).

### Total RNA Extraction and Real-Time PCR Analysis

Total RNA was extracted from wheat grains using RNzol Universal Total RNA Extraction Reagent (Tiangen, Beijing, China). Primers were designed using Primer Premier 6, and TaActin was used as a reference gene (Supplementary Table 1; Ji et al., 2011). RT-PCR analysis was performed using the TransStart Tip Green qPCR SuperMix kit (Transgen, Beijing, China). Each treatment included three biological replicates and three technical replicates. Data were transferred to Excel spreadsheets and analyzed using GraphPad Prism version 6.0 (Livak and Schmittgen, 2001; Zhao et al., 2012; Wang et al., 2017).

### Cloning and Expression of *TaVQ14*

Specific primers were designed to clone the coding sequence of *TaVQ14* (Supplementary Table 1).

The vector pCAMBIA1305 (*p1305*) containing a GPF reporter gene was used for subcellular localization. Primers containing *Xba*I and *Bam*HI restriction sites were designed, and the stop codon was removed to construct the *p1305-CaMV35S-TaVQ14-GFP* expression vector, which served as a control (Dai et al., 2007).

The vector pCAMBIA1301a (*p1301a*) was used in overexpression experiments. Primers containing *Bam*HI and *Xba*I restriction sites were designed, and the *p1301a-TaVQ14* expression vector was constructed.

### Subcellular Localization of TaVQ14

The subcellular localization of TaVQ14 was predicted using CELLO version 2.5 (Yu et al., 2004, 2006). The *Agrobacterium* suspension containing the *p1301a* vector was injected into tobacco leaves, and tissue sections were observed under a confocal microscope.

### Analysis of *TaVQ14* Overexpression in *Arabidopsis*

*Arabidopsis* ecotype Col-0 (WT) was transformed with the p1301-TaVQ14 vector using the floral dip method to obtain mutant T0 seeds. Transformed seeds were screened on MS medium supplemented with hygromycin. SYBR Green I fluorescence quantitative PCR was used to detect the copy number of the exogenously introduced TaVQ14 gene relative to the dxr gene, the dxr gene encoding terpenoid synthase is a single copy in the Arabidopsis genome, setting the internal reference gene (Supplementary Table 1). Positive plants were obtained by cloning GUS gene fragments (Supplementary Table 1) and GUS staining and propagated to T3 overexpressed *Arabidopsis* plants. Seeds from WT and transgenic plants were grown on MS medium supplemented with mannitol (0, 150, or 300 mM) or NaCl (0, 100, or 150 mM) under the same experimental conditions, and the rates of germination were calculated. The calculation formula is: germination rate (GP) = number of normal germinated seeds at the end of germination/number of tested seeds × 100%. The effect of *TaVQ14* overexpression on stress resistance was assessed.

### Analysis of TaVQ14 Homologs

The *atvq15* mutant (SALK_005722), with a mutation in position 17114641 of *AtVQ15* (*AT2G41010*, chr2: 17113798-17115047), was obtained from the Arashare platform. Mutants were screened by three-primer PCR to obtain homozygous plants. Seeds from WT and transgenic plants were grown on MS medium supplemented with mannitol (0, 150, or 300 mM) or NaCl (0, 100, or 150 mM) under the same experimental conditions, and the rates of germination were calculated.

### Measurement of Stress-Related Physiological Indexes and Gene Expression Analysis

Seedlings of WT and transgenic lines were grown in a greenhouse for 24 h in plates containing MS medium supplemented with 300 mM mannitol or 150 mM NaCl, and plates containing MS medium were used as controls. Relative water content (RWC) (Bates et al., 1973; Zhao et al., 2014), and the levels of catalase (CAT) (Rorth and Jensen, 1967; Goldstein, 1968; Del Rio et al., 1977), proline (PRO) (Bates et al., 1973), malondialdehyde (MDA) (Heath and Packer, 1968), superoxide dismutase (SOD) (Sequeira and Mineo, 1966; Kochba et al., 1977), Ascorbate peroxidase (APX) (Zhao et al., 2018), Ascorbic acid (AsA) (Ji et al., 2019), Glutathion reductases (GR) (Zhang et al., 2018), L-Glutathione (GSH) (Xin et al., 2019), and L-Glutathione oxidized (GSSG) (Zhang et al., 2019) were determined, and the expression levels of genes induced by drought stress (*AtRD29A*, *AtRD29B*, *AtP5CS1*, *AtOST1, AtDi19-3, and AtWRKY46*) and salt stress (*AtSHM1*, *AtSOS2*, *AtCDPK2, AtDi19-3, and AtPP2C49*) were quantified (Huang et al., 2012; Qin et al., 2014; Chen J. et al., 2017; Chu et al., 2021).

Seedlings of WT and transgenic lines were grown in square pots for 20 days and treated with 150 mM NaCl or 300 mM mannitol every 3 days. The concentrations of Na^+^, K^+^, and Ca^2+^ were measured on treatment day 12.

### Statistical Analysis

Statistical analysis was performed using SPSS version 19.0 and Origin software.

## Results

### Bioinformatics Analysis of *TaVQ14*

*TaVQ14* has 789 base pairs and encodes a 262-amino acid protein. The protein has a pI of 9.164, MW of 27481.07 Da, hydrophobicity index of 64.39, hydrophilicity index of -0.234, and instability index (II) of 74.16. The percentage of alanine, serine, and proline was 17.9, 14.5, and 11.8%, respectively. In addition, the most common *cis*-acting elements in the promoter region were associated with response to light (64.32%), abscisic acid (21.4%), and auxin (7.14%), and with zein metabolism regulation (7.14%) (Supplementary Table 2).

**FIGURE 1:**
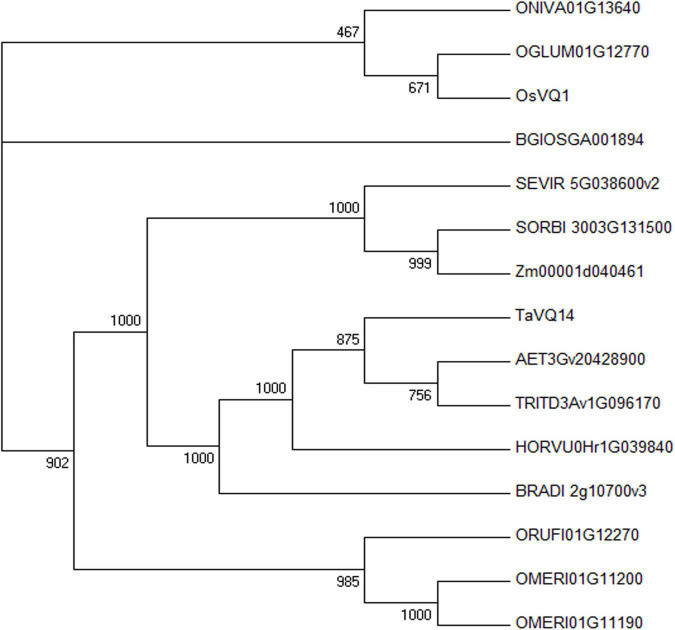
Phylogenetic analysis of *TaVQ14* and its homologs. Homologs*: Aegilops tauschii, AET3Gv20428900; Brachypodium distachyon, BRADI_2g10700v3; Hordeum vulgare, HORVU0Hr1G039840; Oryza glumipatula, OGLUM01G12770; Oryza sativa Indica Group, BGIOSGA001894; Oryza meridionalis, OMERI01G11190; Oryza meridionalis, OMERI01G11200; Oryza nivara, ONIVA01G13640; Oryza rufipogon, ORUFI01G12270; Oryza sativa Japonica Group, OsVQ1 (Os01g0278000); Setaria viridis, SEVIR_5G038600v2; Sorghum bicolor, SORBI_3003G131500; Triticum turgidum, TRITD3Av1G096170; Zea mays, Zm00001d040461.*

Fourteen homologous genes were obtained from the Ensembl Plants database, and a phylogenetic tree was constructed. The results showed that *TaVQ14* was highly homologous with sequences from *Aegilops tauschii, Triticum turgidum, Hordeum vulgare*, and *Brachypodium distachyon* (Supplementary Table 3 and Figure 1).

### Expression Pattern Analysis of *TaVQ14*

**FIGURE 2:**
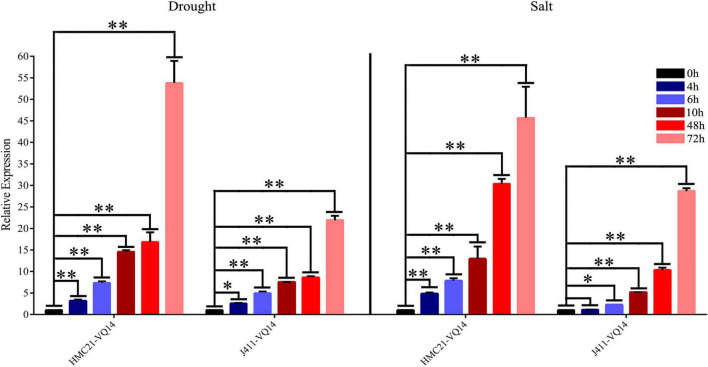
Analysis of *TaVQ14* expression by reverse-transcription quantitative PCR in wheat seeds subjected to drought and NaCl stress. *P < 0.05, **P < 0.01.

Valine-glutamine genes are involved in abiotic stress response in plants (Hu Y. et al., 2013; Kim et al., 2013; Wang M. et al., 2015; Cheng et al., 2020). Our previous study showed that *TaVQ14* was associated with salt stress response (Cheng et al., 2021). Based on these findings, wheat seeds were treated with NaCl or mannitol to assess the role of *TaVQ14* in salinity and drought stress. The results showed that salt and mannitol treatment increased the expression of *TaVQ14* in a time-dependent manner (Figure 2). Furthermore, the expression levels of *TaVQ14* after treatment were similar between the two wheat varieties. These results indicated that *TaVQ14* expression was induced by NaCl and mannitol, suggesting that *TaVQ14* might play essential roles in salinity and drought stress.

### Subcellular Localization Analysis of *TaVQ14*

**FIGURE 3:**
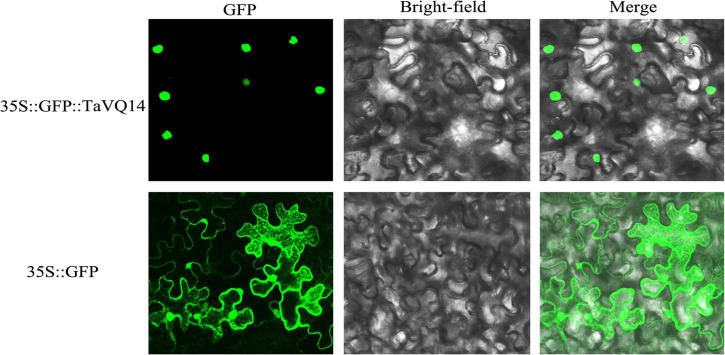
Nuclear localization of TaVQ14. The construct 35S:GFP:TaVQ14 and the control vector 1305 (35S:GFP) were transformed into *Nicotiana tabacum* leaves. The GFP signals in root cells were observed by confocal microscopy.

TaVQ14 was predicted to be found in the nucleus. To confirm this prediction, tobacco leaves were transformed with *Agrobacterium tumefaciens* harboring the *p1305-CaMV35S-TaVQ14-GFP* fusion protein and observed on a confocal microscope. The results showed that the protein signal was detected in the cell nucleus, whereas the GFP control vector was distributed throughout the cell (Figure 3).

### Obtaining of *Arabidopsis* Plants Overexpressing *TaVQ14*

Twenty T1 lines overexpressing *TaVQ14* were obtained. GUS activity results showed that these lines were genetically transformed (stained blue), whereas WT lines (negative control) were not transformed and stained yellow (Supplementary Figure 1a). DNA was extracted from transgenic (T1) and WT plants. The results showed that the transgenic line and positive control, but not the WT line, presented a 650-bp band (Supplementary Figure 1b). We randomly selected five lines from the *TaVQ14* transgenic Arabidopsis positive seedlings to detect the expression level of the target gene *TaVQ14*. The results showed that *TaVQ14* was not expressed in WT, and highly expressed in four lines (Supplementary Figure 1c). In WT, and Lines 1/-2/-3, the copy number of *TaVQ14* gene was detected. The results showed that the *Ct* value of *TaVQ14* gene in WT was greater than 40, indicated that there was no *TaVQ14* gene in this material. The *TaVQ14* gene of Lines 1/-2/-3 were all single copy material (Supplementary Table 4). Transgenic and WT plants were self-crossed for three generations, and their seeds were harvested.

### Resistance to Salt Stress

**FIGURE 4:**
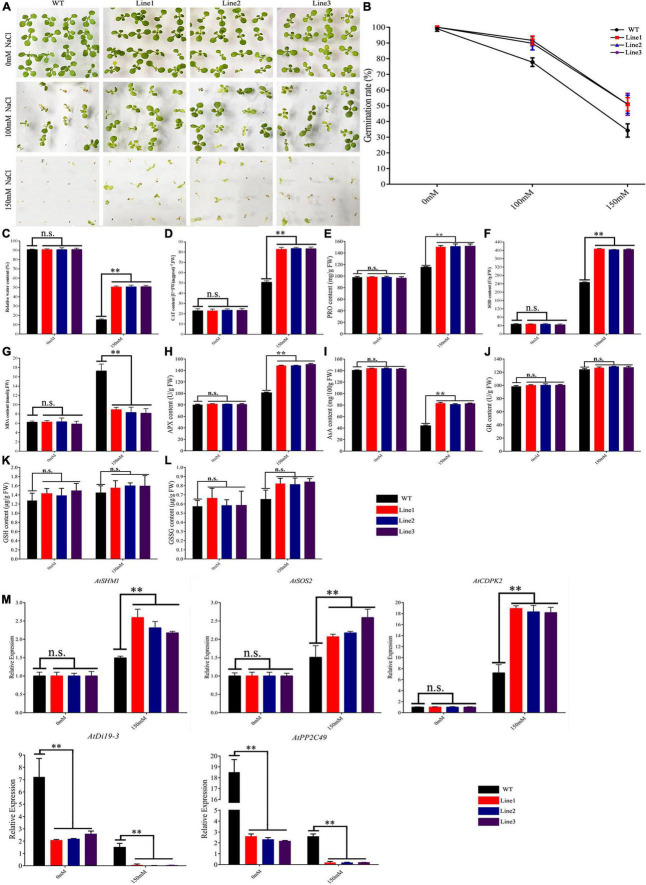
Germination phenotypes of *TaVQ14* in transgenic *Arabidopsis* plants under salt stress. **(A)** Germination performance in *TaVQ14*-overexpressing and wild-type (WT) seeds grown on Murashige and Skoog medium containing 0, 100, or 150 mM of NaCl. **(B)** Rate of germination in transgenic and WT seeds. **(C–L)** Relative water content **(C)** and levels of catalase **(D)**, proline **(E)**, superoxide dismutase **(F)**, malondialdehyde **(G)**, Ascorbate Peroxidase **(H)**, Ascorbic acid **(I)**, Glutathion Reductases **(J)**, L-Glutathione **(K)**, and L-Glutathione oxidized **(L)** in transgenic and WT plants after salt treatment. Values are means ± SE (*n* = 3). *P < 0.05 **P < 0.01 (*t*-test). **(M)** Relative expression levels of salt-responsive genes in transgenic *A. thaliana* plants under normal salinity. Leaves of transgenic and WT plants were collected after salt stress. Y-axis: relative expression levels; X-axis: the time course of stress treatments; Error bars, 6 ± SE.

Seedlings were treated with NaCl (0, 100, or 150 mM), and the rates of germination were calculated. There was no significant difference in germination rate between transgenic and WT plants (100 vs. 99%) before treatment. Treatment with 100 and 150 mM NaCl decreased the rate of germination in WT and transgenic plants; nonetheless, germination was lower in the former (78 and 34% vs. 90 and 51%) (Figures 4A,B).

We measured several physiological indexes in seedlings treated with 150 mM NaCl. Treatment decreased the RWC of transgenic and WT plants; however, the rate of decline was significantly higher in WT plants. Treatment increased the concentrations of CAT, PRO, SOD, APX, GR, GSH, and GSSG in both lines. The increase was more significant in the transgenic line. The increase in MDA levels was less pronounced. The increase in GR, GSH, and GSSG was no obvious changed. Treatment reduced the concentrations of AsA in both lines, the reduce was more significant in WT (Figures 4C–L).

These results showed that transgenic plants had stronger salt tolerance than controls, indicating that the overexpression of *TaVQ14* improved salt tolerance. To further assess the effect of *TaVQ14* on salt tolerance in *Arabidopsis*, we analyzed the expression of salt stress genes (*AtSHM1*, *AtSOS2*, *AtCDPK2, AtDi19-3, and AtPP2C49*) (Huang et al., 2012; Qin et al., 2014; Chu et al., 2021). Salt treatment increased the expressions of *AtSHM1*, *AtSOS2* and *AtCDPK2*, decreased the expression of *AtDi19-3 and AtPP2C49* (Figure 4M). In addition, the relative expression of *AtCDPK2* increased in both groups, suggesting that, as a crucial Ca^2+^ sensor, *AtCDPK2* enhances salt tolerance in *Arabidopsis* seeds.

### Resistance to Drought Stress

**FIGURE 5:**
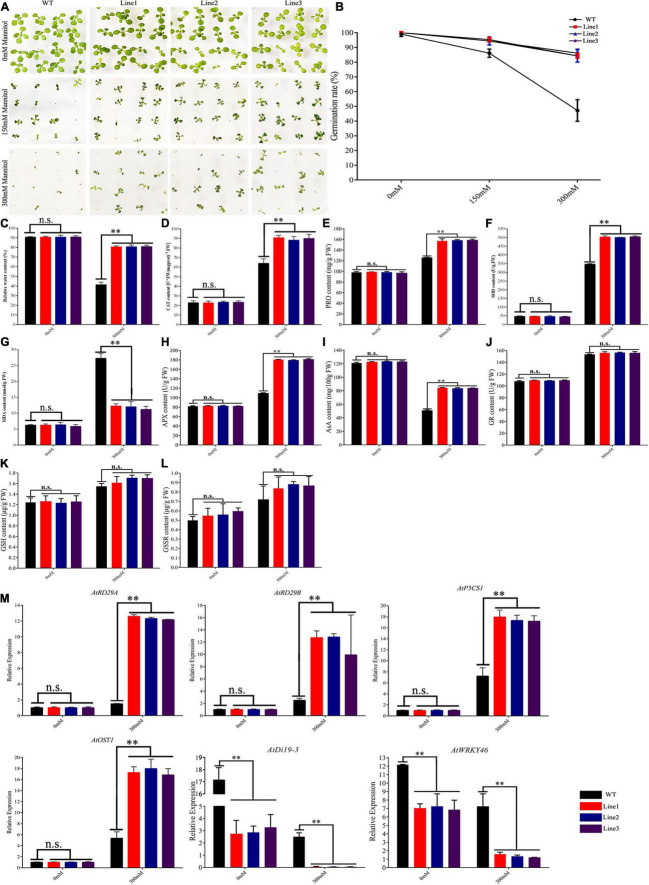
Germination phenotypes of *TaVQ14* in transgenic *Arabidopsis* plants under drought stress. **(A)** Germination performance of *TaVQ14*-overexpressing and wild-type (WT) seeds on Murashige and Skoog medium containing 0, 150, or 300 mM of mannitol. **(B)** Rate of germination in transgenic and WT seeds. **(C–L)** Relative water content **(C)**, and levels of catalase **(D)**, proline **(E)**, superoxide dismutase **(F)**, malondialdehyde **(G)**, Ascorbate Peroxidase **(H)**, Ascorbic acid **(I)**, Glutathion Reductases **(J)**, L-Glutathione **(K)**, and L-Glutathione oxidized **(L)** in transgenic and WT plants after drought treatment. Values are means ± SE (*n* = 3). *P < 0.05 **P < 0.01 (*t*-test). **(M)** Relative expression levels of drought-responsive genes in transgenic *A. thaliana* plants under normal water conditions. Leaves of transgenic and WT plants were collected after drought stress. Y-axis: relative expression levels; X-axis: the time course of stress treatments; Error bars, 6 ± SE.

Seedlings were treated with mannitol (0, 150, or 300 mM), and the rates of germination were measured. There was no significant difference in the percentage of germination between transgenic and WT plants (100 vs. 99%). Treatment with 150 and 300 mM mannitol decreased the rate of germination in both groups; however, germination was less affected in transgenic plants (95 and 85% vs. 86 and 47%) (Figures 5A,B).

Mannitol treatment decreased RWC in both groups, the rate of decline was higher in the WT group. Treatment increased the levels of CAT, SOD, PRO, MDA, APX, GR, GSH, and GSSG. The rate of increase in catalase and SOD was higher in transgenic plants, whereas the rate of increase in MDA was higher in WT plants. The increase in GR, GSH, and GSSG was no obvious changed. Treatment reduced the concentrations of AsA in both lines, the reduce was more significant in WT (Figures 5C–L).

These results showed that transgenic plants had stronger drought tolerance than WT plants, indicating that the overexpression of *TaVQ14* increased drought resistance in *Arabidopsis* seeds. To further evaluate the effect of *TaVQ14* on drought tolerance, the expression levels of drought-related genes (*AtRD29A*, *AtRD29B*, *AtP5CS1, AtOST1, AtDi19-3, and AtWRKY46*) were quantified (Huang et al., 2012; Qin et al., 2014; Chen J. et al., 2017). Drought treatment increased the expression of *AtRD29A, AtRD29B, AtP5CS1*, and *AtOST1*, decreased the expression of *AtDi19-3* and *AtWRKY46* (Figure 5M); suggesting that TaVQ14 improves drought tolerance by enhancing the expression of these genes.

### Measurement of Ion Concentrations

**FIGURE 6:**
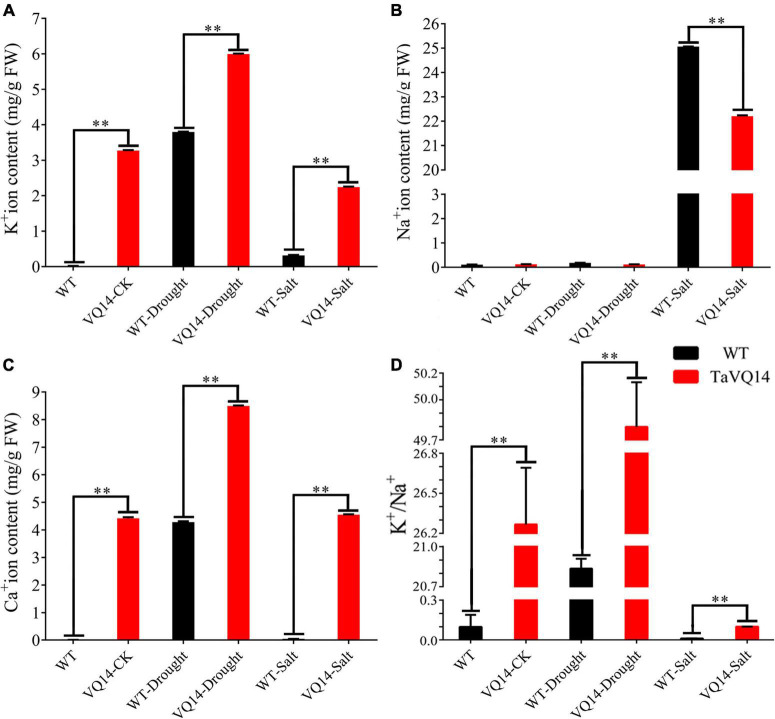
Concentration of Na^+^, K^+^, and Ca^2+^ in transgenic *Arabidopsis thaliana* plants under salt and drought stress. **(A)** K^+^ ion content in transgenic *Arabidopsis thaliana* plants under salt and drought stress. **(B)** Na^+^ ion content in transgenic *Arabidopsis thaliana* plants under salt and drought stress. **(C)** Ca^2+^ ion content in transgenic *Arabidopsis thaliana* plants under salt and drought stress. **(D)** K^+^/Na^+^ in transgenic *Arabidopsis thaliana* plants under salt and drought stress. *P < 0.05, **P < 0.01.

The concentration of K^+^ and Ca^2+^ was significantly higher in *TaVQ14*-overexpressing lines, whereas Na^+^ content was similar between the groups (Figure 6A). Treatment with 150 mM NaCl increased Na^+^ contents in both groups; however, the increase was more pronounced in the WT group (Figure 6B). Treatment with 300 mM mannitol increased K^+^ and Ca^2+^ concentrations in both groups. However, the increase was higher in the transgenic group (Figure 6C).

### Response of *atvq15* Mutants to Salt and Drought Stresses

**FIGURE 7:**
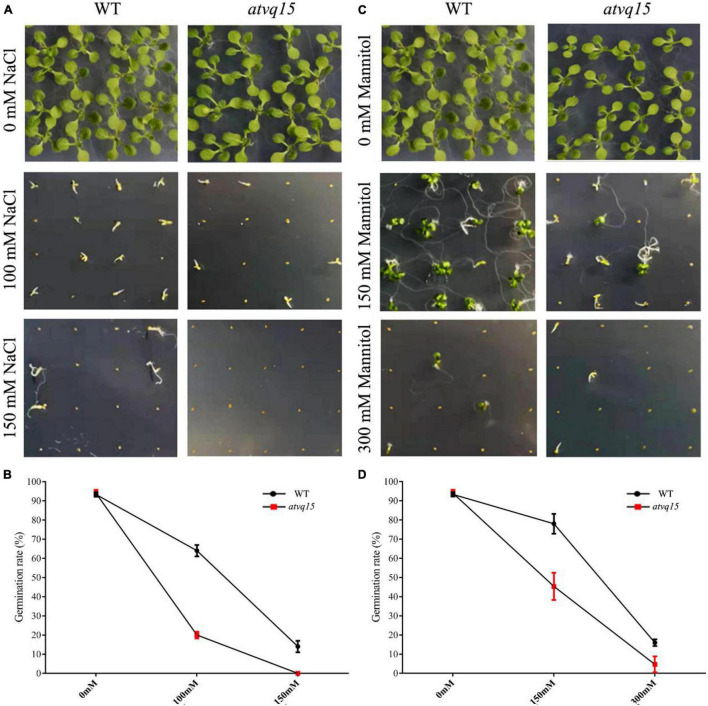
Germination phenotypes of *Arabidopsis thaliana* seeds with a mutation in the gene *atvq15*, a *TaVQ14* homolog, under salt and drought stress. **(A)** Germination performance in *atvq15* mutant and wild-type (WT) seeds grown on Murashige and Skoog (MS) medium containing 0, 100, or 150 mM of NaCl. **(B)** Rate of germination in *atvq15* mutant and WT seeds. **(C)** Germination performance in *atvq15* mutant and WT seeds grown on MS medium supplemented with 0, 150, or 300 mM of mannitol. **(D)** Rate of germination in *atvq15* mutant and WT seeds. Relative water content, and levels of catalase, proline, superoxide dismutase, malondialdehyde, Ascorbate Peroxidase, Ascorbic acid, Glutathion Reductases, L-Glutathione, and L-Glutathione oxidized in atvq14 and WT plants after salt treatment. Relative water content, and levels of catalase, proline, superoxide dismutase, malondialdehyde, Ascorbate Peroxidase, Ascorbic acid, Glutathion Reductases, L-Glutathione, and L-Glutathione oxidized in atvq14 and WT plants after drought treatment.

Homologous genes have similar functions across species (Wang et al., 2017). To further investigate the role of *TaVQ14* homologs in regulating salt and drought resistance, a phylogenetic analysis of *TaVQ14* and VQ family members was performed (Supplementary Figure 2a). The *atvq15* sequence was obtained from the Arashare platform, and detected homozygous plants of *atvq15* mutant by screening leaf DNA (Supplementary Figure 2b). Seeds of *atvq15* mutant and WT plants were treated with mannitol (0, 150, or 300 mM) or NaCl (0, 100, or 150 mM), and the rates of germination were calculated. There was no significant difference in the rate of germination rate between *atvq15* mutant and WT plants (94% in both groups) before treatment (Figure 7). Treatment with 100 and 150 mM NaCl decreased the germination rate in both groups, but the rates were lower in *atvq15* mutants (20 and 0% vs. 64 and 14%) (Figures 7A,B). Treatment with 150 and 300 mM Mannitol decreased the germination rate in both groups; nonetheless, the effect was stronger in *atvq15* mutants (rates of 45 and 5% vs. 78 and 16%) (Figures 7C,D). These results support that *AtVQ15* and *TaVQ14* regulate salt and drought resistance in *Arabidopsis* seeds.

## Discussion

Valine-glutamine proteins are widely found in *Arabidopsis*, rice, maize, soybean, grapes, and other plant species (Cheng et al., 2012; Kim et al., 2013; Li N. et al., 2014; Wang et al., 2014; Wang M. et al., 2015). However, few studies have assessed the functions of these proteins. Wheat is one of the most widely cultivated crops; nonetheless, the functions of *VQ* genes in wheat are incompletely understood. Our previous study has shown that *TaVQ14* encodes an unstable basic hydrophobic protein (Cheng et al., 2021). Therefore, this gene was selected for further functional analysis. Our results showed that *TaVQ14* expression was significantly upregulated under high salinity and drought conditions, indicating that *TaVQ14* was involved in salt and drought stress responses.

*Arabidopsis thaliana* is an excellent model for research in plant biology is an excellent model for research in plant biology, which can obtain transgenic plants in short time. Therefore, transgenic *A. thaliana* lines were used to assess the function of *TaVQ14*. Under drought and salt stress, the rate of germination in *TaVQ14*-overexpressing lines was significantly higher than that of WT plants, indicating that the tolerance of the former to drought and salinity stress was improved.

A plant mutant for *AtVQ15*, a *TaVQ14* homolog, was produced. The results revealed that the percentage of seed germination was lower in these mutants than in WT controls under drought and salt stress. These findings indicate that the *AtVQ15* mutation reduces stress tolerance and that *TaVQ14* and its homolog *AtVQ15* regulate tolerance to drought and salinity. Furthermore, gene expression analysis showed that several genes responsive to drought (*AtRD29A, AtRD29B, AtP5CS1, AtOST1, AtWRKY46*, and *AtDi19-3*) and salinity (*AtSHM1, AtSOS2, AtCDPK2, AtPP2C29*, and *AtDi19-3*) (Huang et al., 2012) were differentially expressed after treatment, suggesting that *TaVQ14* enhances the resistance of *Arabidopsis* seeds to drought and salinity by regulating the expressions of these genes.

The concentrations of K^+^, Na^+^, and Ca^2+^ were measured in *TaVQ14*-overexpressing and WT lines. Ca^2+^ concentration and K^+^/Na^+^ ratio was significantly higher in the transgenic line before treatment, suggesting that *TaVQ14* overexpression improves drought and salt resistance by increasing Ca^2+^ and K^+^ concentrations. Treatment with 300 mM NaCl increased Ca^2+^, K^+^, and K^+^/Na^+^ ratio in the transgenic line, suggesting that *TaVQ14* increases resistance to salt stress by excreting Na^+^ and increasing the uptake of Ca^2+^ and K^+^ (Figures 6A–D). Treatment with 300 mM mannitol increased the uptake of K^+^ and Ca^2+^ in both groups; nonetheless, the increase was more pronounced in the transgenic line, suggesting that *TaVQ14* overexpression improves drought resistance by increasing K^+^ and Ca^2+^ concentrations. *AtCDPK2* was upregulated in the transgenic line under salt stress, suggesting that *TaVQ14* improves salt tolerance by increasing *AtCDPK2* expression through Ca^2+^ signaling. Salinity stress increases cytosolic Ca^2+^ levels. Calcium-dependent protein kinases (CPKs or CDPKs) are strongly implicated in Ca^2+^ signaling in plants and play an important role in salinity stress (Singh et al., 2017). In rice, *OsCPK21* genes regulated the ABA-dependent salt stress signaling pathway (Asano et al., 2011). *OsCPK12* conferred tolerance to salt stress through regulation of ROS homeostasis (Asano et al., 2012). In addition, salinity stress tolerance is stronger in plants overexpressing *OsCPK4* (Campo et al., 2014). These findings reveal that Ca^2+^ signaling, together with ROS signaling and hormonal regulation, mediates the response to salinity stress. However, the mechanisms underlying the regulation of salt tolerance by *TaVQ14* and CDPK need to be further investigated.

Stress resistance is improved by modulating gene expression and physiological and biochemical processes, including the accumulation of osmotic substances and the increase in active oxygen scavenging activity (Zhou et al., 2018; Zhang et al., 2021). Free proline levels in plants are low under normal conditions; nonetheless, under stress conditions, including drought, low temperature, high salinity, and high alkalinity, proline is stored in large quantities, and storage levels are positively correlated with stress resistance. Therefore, proline is used as a biochemical index of stress resistance in plants (Xiang et al., 2007; Hnilickova et al., 2021; Qian et al., 2021; Rajametov et al., 2021). Our results showed that proline level was significantly higher in transgenic plants than in WT controls. Consistent with our results, the large increase in proline concentration increases intracellular osmotic pressure and decreases water potential and water content (Song et al., 2011). MDA is the main product of membrane lipid peroxidation, leading to membrane damage and plant damage. Thus, MDA levels are positively correlated with the degree of membrane lipid peroxidation and can serve as an indicator of cellular reactive oxygen species stress (Xiong et al., 2002; Mittler et al., 2004). Our results showed that MDA concentration was significantly lower in transgenic plants than in WT controls, indicating that *TaVQ14* overexpression increased resistance to oxidative stress. CAT, SOD, and APX are important protective enzymes and reduce oxidative stress by decreasing the production of active oxygen and hydrogen peroxide (May, 2008). In this study, CAT, SOD, and APX levels were significantly higher in the transgenic line than in WT plants, which may explain why the MDA concentration was lower in the former.

The analysis of gene expression, physiological, biochemical, and phenotypic data demonstrated the role of *TaVQ14* overexpression in Arabidopsis improving its salt tolerance and drought tolerance, indicating that *TaVQ14* plays important roles in improving salt tolerance and drought resistance in wheat, and these data provide a basis for the functional analysis of *TaVQ14* in wheat.

China’s Bohai Rim region has more than 40 million mu of medium and low yield farmland and more than 10 million mu of saline alkali wasteland, which has been suffering from drought, waterlogging and alkali disasters for a long time. This experiment proved that *TaVQ14* had the functions of salt tolerance and drought tolerance, and was an excellent salt tolerance and drought tolerance gene. Overexpression and knockout of *TaVQ14* gene in wheat is the direction of our subsequent experimental work, and we hope to widely apply this gene to the cultivation of new wheat varieties with salt tolerance and drought tolerance for the increase of agricultural production and income.

## Data Availability Statement

The original contributions presented in the study are included in the article/Supplementary Material, further inquiries can be directed to the corresponding authors.

## Author Contributions

XC and HY projected the study, put into effect the main bioinformatics analysis, and drew up the manuscript. ZC, CG, and WG carried out the software and helped to handle figures and tables. BT, JJC, and XP participated in the experimental test. SY processed experimental data and joined to amend the manuscript. JJC and XP took part in the software and draw up the manuscript. JL and CXM had a hand in the project of the study and helped to revamp the manuscript. CC and HPZ conceived and guided the experiment, were involved in its project and coordination, and helped to draw up the manuscript. All authors read and accepted the final manuscript.

## Conflict of Interest

The authors declare that the research was conducted in the absence of any commercial or financial relationships that could be construed as a potential conflict of interest.

## Publisher’s Note

All claims expressed in this article are solely those of the authors and do not necessarily represent those of their affiliated organizations, or those of the publisher, the editors and the reviewers. Any product that may be evaluated in this article, or claim that may be made by its manufacturer, is not guaranteed or endorsed by the publisher.

## Footnotes

1
http://gsds.gao-lab.org/

## Abbreviations

VQ: valine-glutamine
ABA: abscisic acid
qRT-PCR: quantitative real-time PCR
RWC: plant water content
MDA: malondialdehyde
CAT: catalase
SOD: superoxide dismutase
PRO: proline
GUS: β -glucuronidase activity
APX: ascorbate peroxidase
AsA: ascorbic acid
GR: glutathion reductases
GSH: l-glutathione
GSSG: L-glutathione oxidized.
