# Supplementary material for: The Wheat Gene TaVQ14 Confers Salt and Drought Tolerance in Transgenic Arabidopsis thaliana Plants
Source: Front Plant Sci. 2022 May 10;13:870586. doi: 10.3389/fpls.2022.870586 (PMC9127792; doi:10.3389/fpls.2022.870586)
Supplement: Supplementary file 1 [file Data_Sheet_1.docx]

**
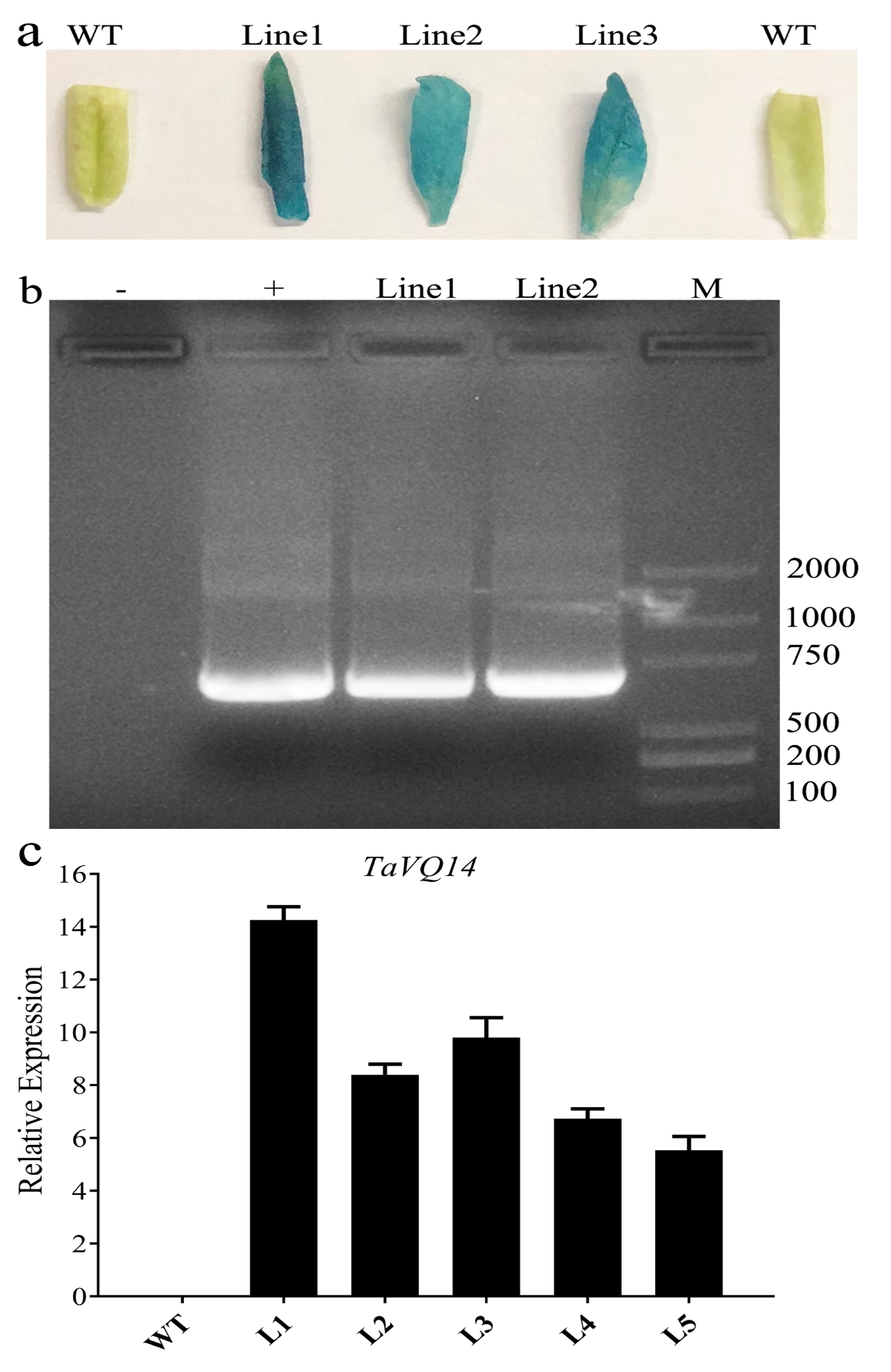
**

**Fig. S1.** Molecular characterization of *Arabidopsis thaliana* plants overexpressing *TaVQ14*.

**a** GUS staining ofTaVQ14 in transgenic and wild-type plants.

**b**^+^Positive control; ^–^Negative control; Lanes 1 and 2: Transgenic *A. thaliana* lines harboring the p1301- *TaVQ14* vector.

**c.** *TaVQ14* expression in overexpress lines and WT


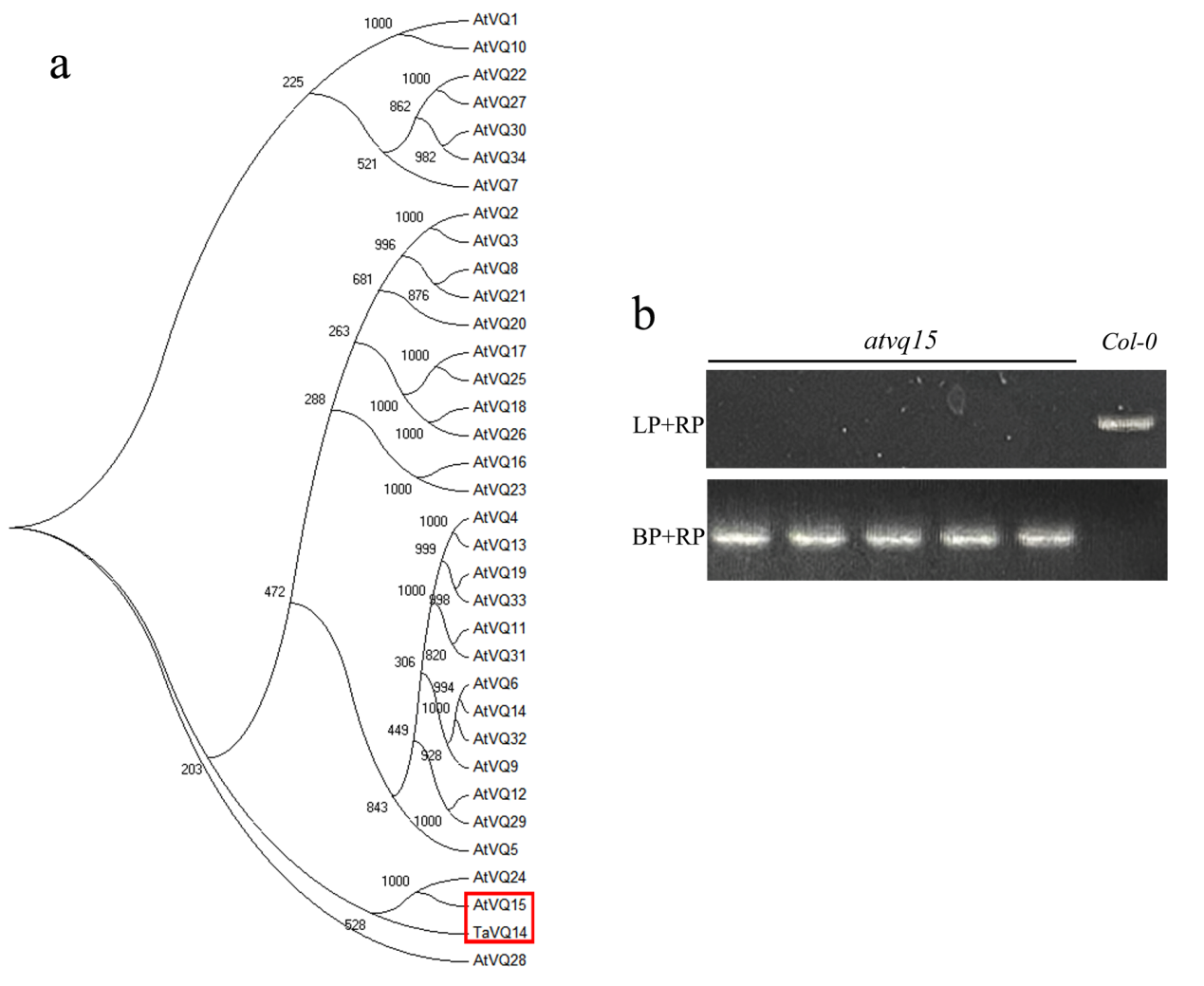


**Fig. S2. a** Phylogenetic analysis of *Arabidopsis thalianaTaVQ14* and other VQ genes.

**b**Electrophoresis analysis of *atvq15* by three-primer PCR.

Table S1. Primer sets.

| Gene | Forward primer | Reverse primer |
| --- | --- | --- |
| Ta-Actin | CCTCTCTGCGCCAATCGT | TCAGCCGAGCGGGAAATTGT |
| TaVQ14-RTPCR | GCAATACGCCGCCTGGTTCTC | GTGGTGGTGGAGGAGGAGGAAG |
| dxr | GTTGTTGCTGCTGCACTTTC | GAGTGTTTCACAGGTGGGTT |
| TaVQ14-Clone | ATGCCGCTCACTTCCTCTTCC | TCAGATCATCCCCCAGCTCTC |
| TaVQ14-1305 | TGCTCTAGAATGCCGCTCACTTCCT | CGCGGATCCGATCATCCCCCA |
| TaVQ14-1301 | TGCGAATTCATGCCGCTCACTTCCT | CGCTCTAGATCAGATCATCCCCCA |
| GUS-Clone | GCGAAGTCTTTATACCGAAAGGTTG | GCCCTTCACTGCCACTGACC |
| At-Actin | AGGCACCTCTTAACCCTAAAGC | GGACAACGGAATCTCTCAGC |
| AtRD29A | ATCACTTGGCTCCACTGTTGTTC | ACAAAACACACATAAACATCCAAAGT |
| AtRD29B | GTGAAGATGACTATCTCGGTGGTC | TACCAAGAGACTCAGCAATCTCTG |
| AtP5CS1 | TTCTCAGATGGTTTCCAGGTTG | TGGGAATGTCCTGATGGGTG |
| AtOST1 | GATCCCGAGGAACCAAAGAA | ATCCTCTTTGCAGGGTCAGC |
| AtSHM1 | AAGGCTTTCTTCTTCAATTG | AGCAATCTCAGGATCAACCT |
| AtSOS2 | ATTGAGGCTGTAGCGAAC | GGTATTCCTTCTGTTGCC |
| AtCDPK2 | GTCCATTACCTTCCCGGCATAT | CAGCAATTACCCGTAATGCCATT |
| AtPP2C29 | GTGTTTCAACAGAGTTTACCGGC | GCTTCAGGCGAAAGACTAAAACAC |
| AtWRKY46 | AGCGAAGCCTTGAGATCGAT | ACTGCCATTAAGAGAGAGAC |
| AtWRKY54 | GATCACATACAAGGATCGTG | AGACCTAGTGCTGATTCATC |
| AtWRKY70 | GATTGGGACCCGTTAAGGGT | CCACTCTACATGGCCTAAT |
| AtDi19-3 | TCTCTTTCAGCTGAGGATCAC | CATGACCTACAAGCAATTGGG |

Table S2 Promoters of TaVQ14

| Function | Element | Number |
| --- | --- | --- |
| ABA | ABRE | 3(21.4%) |
| Auxin | TGA-element | 1(7.14%) |
| Zein metabolism regulation | O2-site | 1(7.14%) |
| Light | G-box | 2(14.28%) |
|  | Sp1 | 5(35.76%) |
|  | TCCC-motif | 2(14.28%) |

Table S3. Orthologs of the *TaVQ14* gene.

| Species | Orthologues |
| --- | --- |
| *Aegilopstauschii* | AET3Gv20428900 |
| *Brachypodiumdistachyon* | BRADI_2g10700v3 |
| *Hordeum vulgare* | HORVU0Hr1G039840 |
| *Oryzaglumipatula* | OGLUM01G12770 |
| *Oryza sativa Indica Group* | BGIOSGA001894 |
| *Oryzameridionalis* | OMERI01G11190 |
| *Oryzameridionalis* | OMERI01G11200 |
| *Oryzanivara* | ONIVA01G13640 |
| *Oryzarufipogon* | ORUFI01G12270 |
| *Oryza sativa Japonica Group* | OsVQ1(Os01g0278000) |
| *Setariaviridis* | SEVIR_5G038600v2 |
| *Sorghum bicolor* | SORBI_3003G131500 |
| *Triticumturgidum* | TRITD3Av1G096170 |
| *Zea mays* | Zm00001d040461 |
|  |  |

Table S4. Copy number of *TaVQ14* and *dxr* gene in different transgenic lines

|  | *TaVQ14* | | *dxr* | | Ratio to *dxr* | Average value | Copy number of *TaVQ14* gene |
| --- | --- | --- | --- | --- | --- | --- | --- |
|  | Ct value | Amount of template | Ct value | Amount of template |  |  |  |
| Line1 | 23.0250 | 0.3483*C | 28.4567 | 0.2546*C | 1.3680 | 1.4615 | 1 |
|  | 22.7846 | 0.3951*C | 28.5701 | 0.2415*C | 1.6360 |  |  |
|  | 22.9908 | 0.3546*C | 28.4374 | 0.2569*C | 1.3803 |  |  |
| Line2 | 24.5213 | 0.1589*C | 29.9860 | 0.1249*C | 1.2722 | 1.1402 | 1 |
|  | 24.9849 | 0.1246*C | 30.1521 | 0.1156*C | 1.0779 |  |  |
|  | 24.7051 | 0.1443*C | 29.8222 | 0.1348*C | 1.0705 |  |  |
| Line3 | 27.4114 | 0.0349*C | 33.3643 | 0.0259*C | 1.3475 | 1.0457 | 1 |
|  | 29.1944 | 0.0137*C | 33.9410 | 0.0198*C | 0.6919 |  |  |
|  | 27.1850 | 0.0393*C | 32.6692 | 0.0358*C | 1.0978 |  |  |
| WT | 42.9830 | 0*C | 33.3643 | 0.0259*C | 0 | 0 | 0 |
|  | 40.5930 | 0*C | 33.0132 | 0.0305*C | 0 |  |  |
|  | 40.9030 | 0*C | 33.4061 | 0.0254*C | 0 |  |  |
